# Janus Kinase Inhibitor Use Among People With Inflammatory Disease in Australia

**DOI:** 10.1002/pds.70503

**Published:** 2026-10-30

**Authors:** Claire T. Deakin, Michael O. Falster, Geoffrey O. Littlejohn, Peter Youssef, David Liew, Nicole L. Pratt, Sallie‐Anne Pearson

**Affiliations:** ^1^ Medicines Intelligence Centre of Research Excellence, School of Population Health, Faculty of Medicine and Health, UNSW Sydney Sydney Australia; ^2^ Department of Medicine Monash University Clayton Victoria Australia; ^3^ Royal Prince Alfred Hospital Sydney New South Wales Australia; ^4^ Faculty of Medicine and Health University of Sydney Sydney New South Wales Australia; ^5^ Institute for Musculoskeletal Health, Sydney Local Health District Sydney New South Wales Australia; ^6^ Department of Rheumatology Austin Health Heidelberg Victoria Australia; ^7^ Department of Clinical Pharmacology and Therapeutics Austin Health Heidelberg Victoria Australia; ^8^ Department of Medicine University of Melbourne Parkville Victoria Australia; ^9^ Quality Use of Medicines and Pharmacy Research Centre, Clinical and Health Sciences, University of South Australia Adelaide Australia

**Keywords:** b/tsDMARD, biological therapy, inflammatory diseases, JAK inhibitors, pharmacoepidemiology, small molecule inhibitor, targeted therapy

## Abstract

**Aim:**

To describe Australian trends in JAKi uptake, where there are no restrictions on the sequence of their use in clinical practice.

**Methods:**

We included all people aged 18 years and over initiating a JAKi between 1 October 2015 and 31 August 2022. We used national dispensing claims data for a random 10% sample of Australians to: describe the number of people initiating JAKi per quarter; the characteristics of new JAKi users including demographics, use of other medicines to manage cardiovascular comorbid disease; and use of prior targeted therapy (biologics or small molecule inhibitors). We used Kaplan–Meier analysis to estimate the proportion of initiators who were persistent on their initial JAKi to 365 days.

**Results:**

We identified 2904 people initiating JAKi treatment between 1 October 2015 and 31 August 2022; new use increased consistently during the study period. 45% of initiators had not been dispensed a prior subsidised targeted therapy in 365 days prior to JAKi initiation; 72% of new users were female and 62% were aged 55 years or older. Almost half of people initiating JAKi had at least one marker of higher cardiovascular risk, with 30% dispensed medicines to treat or prevent cardiovascular disease in the year prior to initiation; and 34% being aged 65 years or older. 60% of people persisted on their initial JAKi therapy to 365 days. Of those who stopped, 51% switched to a biologic and 15% switched to a different JAKi.

**Conclusion:**

In a setting where JAKi are permitted to be used in any line of therapy, 45% of new users of JAKi were naïve to targeted therapy and half had at least one marker of cardiovascular risk. Continued monitoring is required to understand the risk profile of JAKi, particularly in those with high baseline cardiovascular disease risk.

## Five Key Points

- JAKi are effective for treating inflammatory diseases including arthritis, bowel disease and dermatitis; variations in prescribing restrictions for subsidised access to targeted therapy (biologics or small molecule inhibitors) mean the place in therapy of JAKi differs across health systems, indications and stage of disease.
- Real‐world evidence is emerging on JAKi use across global jurisdictions, providing insights supporting policy maker, prescriber, and patient decision making as they continue to evaluate the population‐ and individual‐level risks and benefits of these therapies.
- We observed increasing uptake of JAKi in Australia between 1 October 2015 and 31 August 2022 with nearly half of people initiating JAKi naïve to targeted therapy; most people initiating JAKi were dispensed medicines indicated for rheumatoid arthritis.
- Nearly half of people initiating JAKi had markers of increased cardiovascular risk, being over 65 years of age or dispensed medicines to treat or prevent cardiovascular disease in the year prior to JAKi initiation.
- 60% of people initiating JAKi persisted on treatment for at least 365 days, indicating they likely tolerated and responded to JAKi therapy.

## Plain Language Summary

This research investigated how janus kinase inhibitors (JAKi) were used in Australia. JAKi are a class of medicine used to treat autoimmune and inflammatory conditions such as rhematoid arthritis. JAKi may offer better control of disease symptoms, but their long‐term safety is uncertain. A recent safety trial reported a link between JAKi and cardiovascular disease. Australia is a useful setting for understanding JAKi use, because there are no restrictions on the order in which JAKi or other targeted therapies can be used. This study used a 10% sample of the Australian population between 1 October 2015 and 31 August 2022. From 1 October 2015 until 31 August 2022, there were 2904 people who started a JAKi medicine and almost half of them had not been treated with a targeted therapy during the year beforehand. Most people stayed on treatment for at least 1 year. Almost half of people who started using a JAKi medicine had at least one marker of cardiovascular risk. These findings, and ongoing monitoring, will build knowledge about the safety of JAKi.

## 1 Purpose

Janus kinase inhibitors (JAKi) are a class of targeted synthetic small molecule inhibitors (SMI) used to treat people with inflammatory joint, skin and bowel diseases who have not responded to conventional immunosuppressant medicines such as methotrexate, corticosteroids and aminosalicylates. Randomised clinical trials have demonstrated superior efficacy of the JAKis tofacitinib, baricitinib and upadacitinib for several indications compared to conventional immunosuppressants or biologics [1, 2, 3, 4, 5, 6, 7, 8, 9]. Observational studies have demonstrated JAKi have similar treatment effectiveness (measured by treatment duration and/or discontinuation rates) compared to biologics such as tumour necrosis factor inhibitors (TNFi) [10, 11, 12, 13, 14]. However, most of these studies focused solely on JAKi as second or later line treatment for rheumatoid arthritis, after treatment failure on a biologic.

In Australia, JAKi are publicly subsidised on the Pharmaceutical Benefits Scheme (PBS) for people with moderate to severe disease who have not responded to prior therapy. There are no PBS restrictions on the order of targeted therapy in which JAKi can be prescribed. In contrast, jurisdictions including the UK and Scandinavia only allow a JAKi to be prescribed after treatment failure with a biologic. In the US, access to targeted therapies from major public schemes, such as Medicare, require copayments ranging from USD $2000 to $5200 [15]. These costs can be prohibitively high for the people eligible for these schemes, such as those aged 65 years or older or with disabilities. Australia's policy environment provides a unique opportunity to monitor the real‐world use of JAKi in a setting free from sequencing restrictions and significant cost barriers for access.

To date, no peer‐reviewed publications have examined JAKi use across multiple indications in an unrestricted setting. Existing literature has focused on specific indications such as rheumatoid arthritis, psoriatic arthritis and atopic dermatitis [16, 17, 18, 19, 20]. Against this backdrop, emerging safety concerns have prompted global regulatory scrutiny since 2019 [21, 22, 23, 24, 25, 26]. In 2023 Australia's Therapeutic Goods Administration (TGA) issued warnings about the potential risks associated with JAKi use, including serious cardiovascular events and malignancies, underscoring the need to better understand real‐world use and the clinical characteristics of JAKi users.

The aim of this study was to describe the real‐world use of all JAKi in Australia between 2015 and 2022, before the TGA safety warnings. Specifically, we examined trends in the JAKi incidence, use in people naïve to targeted therapy, concomitant medicine use and characteristics of JAKi initiators including markers of increased cardiovascular risk, and discontinuation and/or switching of therapy within 365 days.

## 2 Methods

### 2.1 Setting

Australian citizens and permanent residents can access prescription medicines subsidised by the Australian government under the Pharmaceutical Benefits Scheme (PBS). People contribute a fixed co‐payment towards their medicine costs depending on their entitlement to concessions, with lower co‐payments accessed by pensioners, low‐income earners and people with a disability. In 2015–2022 the co‐payment thresholds were up to $6.80 for concession card holders and up to $42.50 otherwise for Australian citizens and residents.

### 2.2 Data Source

The Australian Government Services Australia routinely collects data on dispensings subsidised under the PBS. In this study, we used the PBS 10% sample, which is a deidentified and longitudinal extract of dispensing records for a 10% random sample of PBS‐eligible persons [27]. This is a source of routinely collected data which has been used for multiple pharmacoepidemiology studies, such as analyses of post‐market medicine use and the impact of medicines policies [28, 29, 30, 31]. The PBS 10% sample includes information about the Anatomic Therapeutic Chemical (ATC) code of the medicine dispensed, a PBS item code for each medicine, the date of supply, the date of prescription, the specialty of the prescriber, and the sex, year of birth year of death and a unique identifier for each person. All dispensing dates are offset by up to 14 days and all years of birth and death are offset by up to 6 months to protect privacy. Medicines dispensed privately without a PBS subsidy or medicines dispensed to public hospital in‐patients are not captured in the PBS 10% sample.

The PBS 10% sample does not contain direct information about the indication for prescribing medicines. PBS item codes represent medicine formulations available for a claim under the PBS. Authority codes are used for medicines on the PBS, including high‐cost targeted therapies (biologic medicines and small molecule inhibitors (SMI)), which can only be prescribed for approved indications after the prescriber has requested authority from the Australian Government Services Australia.

### 2.3 Medicines of Interest

PBS subsidised JAKi and other targeted therapies are available for people with moderate to severe disease for: rheumatoid arthritis, psoriatic arthritis, ankylosing spondylitis, non‐radiographic axial spondyloarthritis, psoriasis, ulcerative colitis, atopic dermatitis, Crohn's disease and juvenile idiopathic arthritis (Figure 1).

**FIGURE 1:**
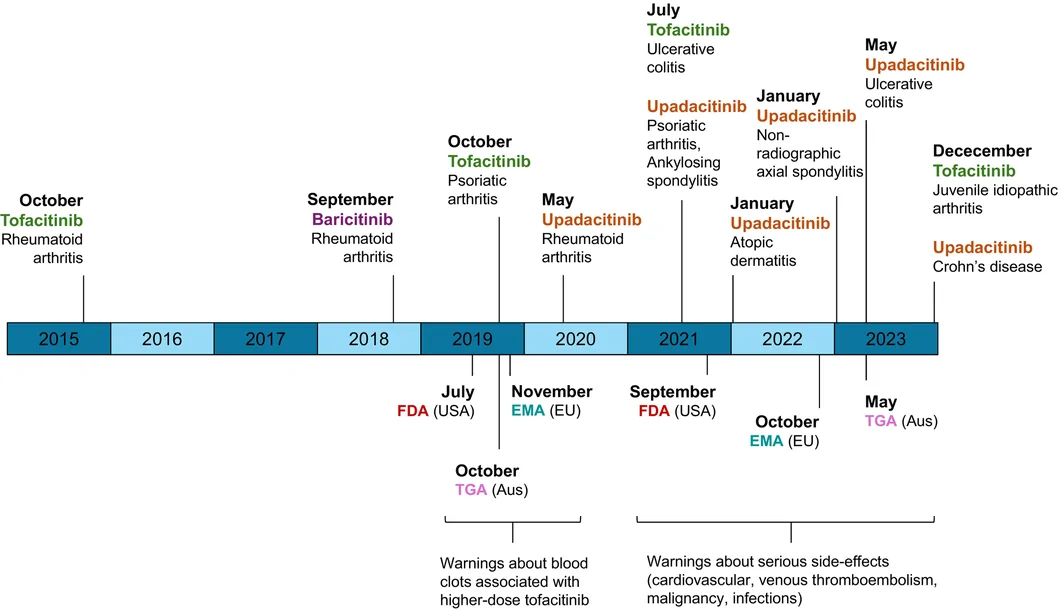
Timeline of JAKi listings on Australia's Pharmaceutical Benefits Scheme for each indication and safety warnings issued by regulatory authorities. Aus, Australia; EMA, European Medicines Agency; EU, European Union; FDA, Food and Drug Administration; TGA, Therapeutic Goods Administration; USA, United States of America.

There are three PBS‐listed JAKi medicines: tofacitinib (ATC code L04AA29), baricitinib (ATC code L04AA37) and upadacitinib (ATC code L04AA44). Tofacitinib was the first PBS‐listed JAKi and was subsidised for rheumatoid arthritis in October 2015, with baricitinib and upadacitinib becoming available for rheumatoid arthritis in September 2018 and May 2020, respectively (Figure 1). PBS item codes and ATC codes for each indication for tofacitinib, baricitinib and upadacitinib available within our study period (1 October 2015 to 31 August 2023) are described in Table S1.

### 2.4 Study Design and Population

We performed a retrospective cohort study. We included all people aged 18 years and over initiating JAKi medicines, defined new use as no dispensings of any JAKi in the prior 365 days, from 1 October 2015 to 31 August 2022. Our study period coincides with the entry of JAKi to the Australian market and there were no publicly subsidised dispensings of JAKi prior to this. Most of these people are likely to have been prescribed JAKi for rheumatoid arthritis, as the first indication for subsidised JAKi in Australia and the most prevalent of the JAKi indications [32]. People who initiated JAKi were followed up for 365 days to examine treatment persistence. People were eligible to enter the cohort once when they initiated their first JAKi. People who initiated a subsequent JAKi did not re‐enter the cohort. A diagram of study design is presented in Figure S1.

### 2.5 Trends in JAKi Initiation

We described quarterly trends in JAKi initiation and defined the date of initiation as the first date of JAKi supply. We also reported quarterly trends of initiation where people were naïve to a prior biologic medicine, defined as people who had not been dispensed a biologic medicine in the 365 days prior to initiating JAKi. As a sensitivity analysis, we tested an alternative definition of biologic‐naïve JAKi initiators as people who had not been dispensed a biologic medicine in the 5 years prior to initiating JAKi. We stratified quarterly trends by the indication for JAKi (derived from PBS item codes) and stratified by the first specific JAKi medicine that was initiated (tofacitinib, baricitinib or upadacitinib). It is possible that there may be some indication misclassification as the same product can be used for multiple indications and at the time of dispensing may be assigned an incorrect PBS item code. To determine the validity of inferring indication by PBS item code we also described the specialty of prescribers for the PBS item codes dispensed to JAKi new users in Table S2. While over 90% of dispensings were prescribed by an appropriate specialist for the JAKi indications, we acknowledge that we cannot infer the indication for JAKi using the PBS item codes with complete certainty in these data.

### 2.6 Characteristics of New Users

We described the characteristics of people initiating JAKi at the date of initiation, including age, sex and concomitant medicines, including anti‐inflammatory medicines such as corticosteroids, disease‐modifying antirheumatic drugs (DMARDs), aminosalicylates and non‐steroidal anti‐inflammatory drugs (NSAIDs) (Table S3). We described antiviral medicine use (Table S4). We did not include targeted therapies in the definition of concomitant anti‐inflammatory medicines. The PBS 10% sample does not contain information about the duration of treatment, so we defined concomitant medicine use as follows. First, we calculated the number of days between dispensings of the same PBS item code. We then determined the 75% percentile of the number of days between dispensings, as an estimated period of supply for each dispensing. A medicine was then determined to be concomitant if it was dispensed on the date of JAKi initiation or within that 75th percentile dispensing interval prior to JAKi initiation.

We also described medicine use for selected comorbidities within 365 days prior to JAKi initiation. We calculated the Rx‐Risk Comorbidity Index [33], which estimates comorbidity burden based on medicines used to manage comorbidities, within 365 days before JAKi initiation. Specifically, we used the Rx‐Risk categories of hypertension, hyperlipidaemia, anticoagulants and antiplatelets to indicate people dispensed medicines to treat or prevent cardiovascular conditions (Table S5). The Rx‐Risk has been validated as a reasonable proxy for cardiovascular disease identification in a recent analysis of Australian linked administrative data, which compared conditions identified using the Rx‐Risk to both self‐reported conditions and diagnoses recorded using ICD‐10‐AM during hospitalisation [34]. We identified people with markers of cardiovascular risk at JAKi initiation as being aged over 65 years, or dispensed at least one medicine to treat or prevent cardiovascular conditions. The PBS 10% sample does not include other potential risk factors such as smoking status or risk of cancer.

### 2.7 Persistence of JAKi Use to 365 Days

We analysed persistent use of JAKi at 1 year, defined as continued use of a specific JAKi at the level of ATC code without a gap in treatment from the first dispensing of that JAKi to 365 days follow‐up. We estimated the period of exposure to a specific formulation of a JAKi, defined by a PBS item code, using the 75th percentile of days between dispensings of the same JAKi PBS item code, excluding time periods greater than 180 days (Table S6) [35]. Similar to our previous work, we defined a gap in treatment as three times the estimated period of exposure from the last dispensing record for the ATC code for the specific JAKi [36, 37]. We defined treatment discontinuation as the date of the last dispensing record for the ATC code plus the estimated period of exposure. We used a sensitivity analysis to test the impact of an alternative definition of treatment discontinuation based on shorter gap in treatment i.e., two times the estimated period of exposure. We did not include information about dose in our calculation, as a higher strength formulation had limited availability within our study period (10 mg twice daily dose of tofacitinib indicated for ulcerative colitis). We described the class of targeted therapy people were switched to after JAKi (TNFi, a different JAKi, other non‐TNFi biologic). We followed people until treatment discontinuation, date of death or until 365 days after the date of JAKi initiation. Since the date of death is offset by up to 6 months in the PBS 10% sample, we used a proxy date of death based on the last dispensing of any medicine within the possible window for the date of death [36]. We censored people at the date of death, treatment discontinuation or 365 days, whichever was earliest.

### 2.8 Statistical Analysis

We summarised categorical data using counts and percentages and continuous data using median and interquartile range (IQR). We used Kaplan–Meier analysis to estimate time to first JAKi discontinuation.

All analyses were performed using R version 4.3.1 [38]. We used the R package ‘phamacoepiToolbox’ version 0.1.0 to calculate persistence as described above [37]. We used the ‘arrow’ (version 13.0.0.1) and ‘parquetize’ (version 0.5.6.1) packages for data management and the ‘survival’ package (version 3.5–5) for Kaplan–Meier analysis [39, 40, 41].

## 3 Results

### 3.1 Trends in JAKi Initiation

The 2904 people in the PBS 10% sample initiated JAKi treatment between 1 October 2015 and 31 August 2022; with new use increaing by over 3‐fold during the study period (Figure 2). Most new users were using JAKi for rheumatoid arthritis. The rate of initiation of JAKi subsidised for rheumatoid arthritis stabilised from 2019 (Figure S2). All JAKi initiations up to June 2018 were tofacitinib and by September 2020, upadacitinib constituted approximately 50% of JAKi initiations. Almost half of the people initiating JAKi had not been dispensed a prior PBS‐listed targeted therapy in the year prior to JAKi initiation. This proportion was consistent during the study period and remained high in our sensitivity analyses using a more stringent definition targeted therapy‐naïve (no dispensings within 5 years prior to JAKi initiation; Figure S3).

**FIGURE 2:**
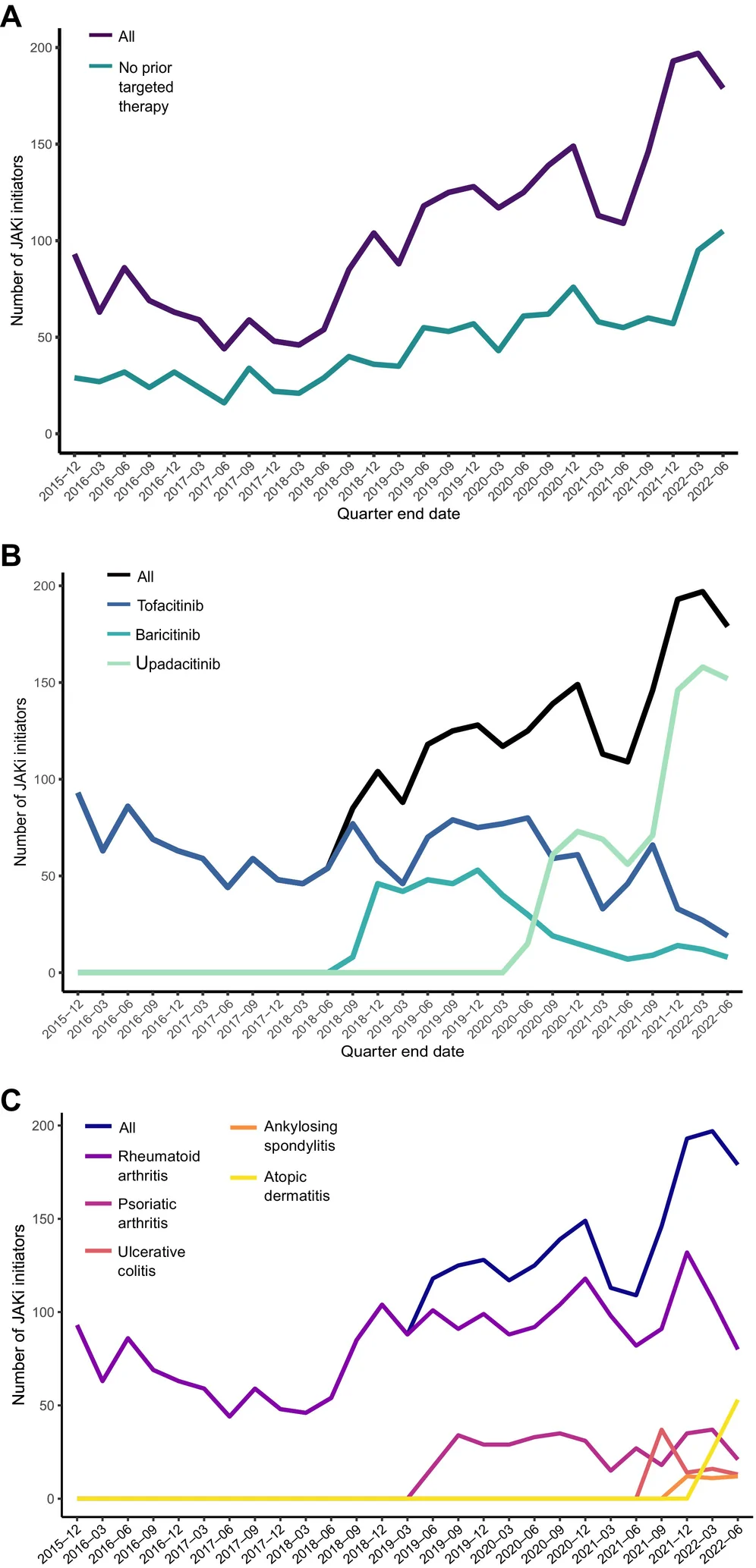
Quarterly temporal trends of JAKi initiation in Australia stratified by (Panel A) targeted therapy new user status, (Panel B) JAKi medicine, and (Panel C) indication. New users naïve to targeted therapy were defined as no prior biologic or SMI therapy within 365 days prior to JAKi initiation.

### 3.2 Characteristics of New Users of JAKi

Approximately three‐quarters of people initiating JAKi for any indication were female (Table 1). 2133 (62.2%) were aged 55 years or older, and 971 (33.4%) were aged 65 years or older. These demographic characteristics reflect the high proportion of new users of JAKi who had rheumatoid arthritis (Table S6). Over 50% of initiators were dispensed tofacitinib, and 30% were dispensed upadacitinib.

**TABLE 1:** Characteristics of people initiating JAKi.

	*n* = 2904
Sex (*n*, %)	
Female	2094 (72.1%)
Male	810 (27.9%)
Age at initiation, years (median, IQR)	59 [49–68]
Age group at initiation (*n*, %)	
18–34 years	215 (7.4%)
35–44 years	305 (10.5%)
45–54 years	578 (19.9%)
55–64 years	835 (28.8%)
65–74 years	666 (22.9%)
75–84 years	275 (9.5%)
85 years and older	30 (1.0%)
JAKi medicine at initiation (*n*, %)	
Tofacitinib	1606 (55.3%)
Baricitinib	417 (14.4%)
Upadacitinib	881 (30.3%)
Initiation of high dose tofacitinib, 10 mg twice daily (*n*, %)	72 (2.5%)
Prescriber specialty at JAKi initiation	
Rheumatology	2045 (70.4%)
Gastroenterology	91 (3.1%)
Dermatology	106 (3.7%)
Immunology	82 (2.8%)
General medicine	159 (5.5%)
Other or not recorded	421 (14.5%)
Concomitant PBS medicine use at JAKi initiation (*n*, %)	
None	696 (24.0%)
Immunosuppressant	2252 (77.5%)
Corticosteroid	2068 (71.2%)
NSAID	1296 (44.6%)
Number of unique targeted therapies dispensed up to 365 days prior (*n*, %)	
0	1296 (44.6%)
1	1258 (43.3%)
2	300 (10.3%)
≥ 3	383 (13.2%)
Class of most recent targeted therapies dispensed within 365 days prior (*n*, %)	
TNFi	970 (60.3%)
Other biologic	638 (39.7%)
Medicine use for comorbidities up to 365 days prior (*n*, %)	
Cardiovascular disease or risk (at least one of the following)	812 (28%)
Hypertension	595 (20.5%)
Hyperlipidaemia	785 (27.0%)
Anticoagulants	237 (8.2%)
Antiplatelets	110 (3.8%)
Diabetes	260 (9%)
Hepatitis B, hepatitis C, HIV or tuberculosis	148 (5.1%)
Malignancies	25 (0.9%)
Renal disease	15 (0.5%)
Anxiety, bipolar disorder, depression or psychotic illness	1102 (37.9%)
Rx‐Risk comorbidity index (median, IQR)	4 [2–6]
Antiviral use up to 5 years prior (*n*, %)	0 (0%)
Aged over 65 years and medicine use for cardiovascular disease or risk	453 (15.6%)

Over half of JAKi initiators had received a prior targeted therapy, with TNFi being the most common class; most (76%) had a concomitant dispensing of another anti‐inflammatory medicine.

The 1330 people (45.8%) had at least one of marker of increased cardiovascular risk; 33% were aged 65 years or older and 28% of JAKi initiators had received medicines to treat or prevent cardiovascular disease in the year prior to initiating JAKi, the most frequent being statins and antihypertensives (Table 1). There were 453 people (15.6%) who had both markers of risk (Table 1); this proportion was highest among people using medicines for rheumatoid arthritis (Table S7).

### 3.3 Persistence to JAKi

Of the 2904 people initiating JAKi in the PBS 10% sample, 60% were persistent at 365 days (Table 2; Figure 3). This finding remained in our sensitivity analyses testing a more stringent definition of treatment persistence (Table S8). Of those who stopped treatment with their initial JAKi product, 51% switched to a biologic, while 15% switched to a different JAKi. Persistence to JAKi was highest for upadacitinib (67%), then baricitinib (62%), then tofacitinib (55%; Table S9, Figure S4). Persistence was similar across age groups (Table S9, Figure S5).

**TABLE 2:** Persistent JAKi use to 365 days and switching of targeted therapy after JAKi cessation.

	JAKi initiators (*n* = 2904)
Persistent JAKi use to 365 days	1735 (59.7%)
Subsequent targeted therapy received within 365 days after JAKi cessation (*n* = 1169)^a^	
No targeted therapy (stopped treatment)	396 (33.9%)
TNFi	338 (28.9%)
Other biologic	263 (22.5%)
Other JAKi	172 (14.7%)

^a^ Denominator for subsequent targeted therapy received is 1169 JAKi users who stopped JAKi treatment within 365 days.

**FIGURE 3:**
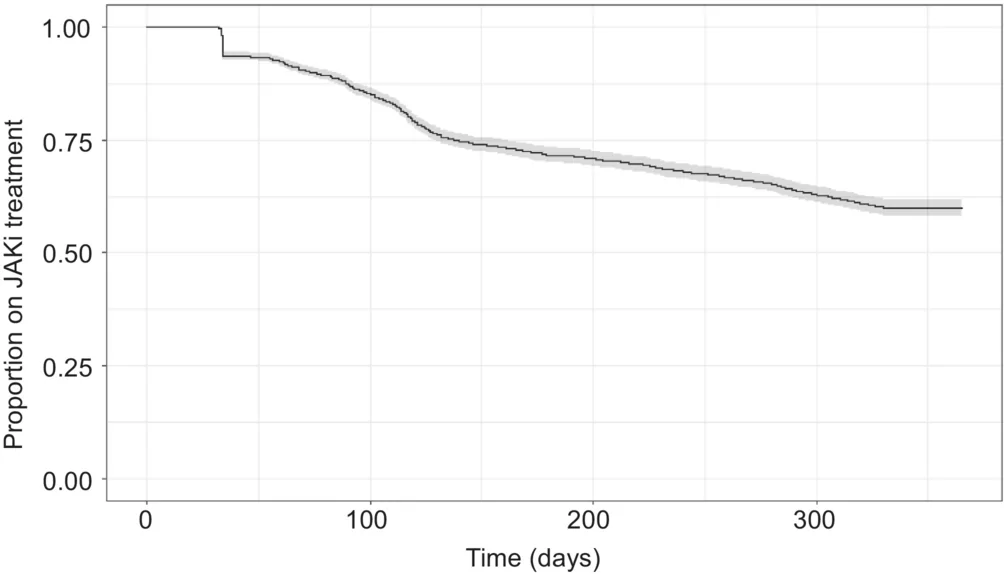
Kaplan–Meier analysis of persistence to JAKi to 365 days for people who initiated JAKi between 1 October 2015 and 31 August 2022.

Of the 1296 who were naïve to prior targeted therapy, 820 (63%) were persistent at 365 days. The 68 of 476 who stopped treatment with their initial JAKi switched to another JAKi, 47 switched to another biologic and 136 switched to TNFi.

## 4 Discussion

We observed increasing uptake of JAKi over time, with high uptake as first‐line targeted therapy. While most people remained on treatment for at least 1 year, half of those who did so switched to a biologic which may suggest either treatment failure or potential adverse effects. The trend of increasing new use likely reflects the entry of new JAKi such as upadacitinib into the Australian market for rheumatoid arthritis as well as an expansion of the indications for JAKi. These findings are consistent with an analysis of treatment patterns for people with rheumatoid arthritis before and after JAKi entered the Australian market, which found use of TNFi decreased from 63% to 46% and use of other biologics decreased from 36% to 26% [42]. Our findings differ from international observational studies which have reported on JAKi use in settings where JAKi are less frequently used by people who have not tried biologic therapy [43, 44]. However, our findings are consistent with the large European JAK‐pot multi‐register study, which reported 31.6% of JAKi initiators with rheumatoid arthritis had no prior targeted therapy [10]. In that study, people initiating JAKi had a mean age of 58.2 years, 81.1% of them were female and the median JAKi persistence was 1.19 years.

It will be important to monitor trends in JAKi uptake particularly as information about JAKi safety emerges. Since the ORAL Surveillance post‐market trial reported higher risks of major adverse cardiac events (MACE), cancer and infections associated with JAKi compared to TNFi in people with rheumatoid arthritis, aged over 50 years with at least one cardiovascular risk factor [45]. In response, regulatory authorities globally, including Australia's Therapeutic Goods Administration (TGA), have issued safety warnings for people using these medicines with specified risk factors including cardiovascular disease, cancer and age over 65 years [21, 22, 23, 24, 25, 26]. Our study was undertaken prior to the TGA warning [25, 26]. We found almost half of initiators had at least one marker of cardiovascular risk. This finding reflects the elevated cardiovascular risk for people with systemic inflammatory conditions and the older age of the cohort [46]. Previous analyses of JAKi and TNFi use for rheumatoid arthritis have described similar proportions of cardiovascular disease among JAKi and TNFi initiators, but JAKi initiators were older [47, 48]. The timing of the TGA warning may also account for the high proportion of initiation of JAKi in people who had not been dispensed a prior targeted therapy, as the TGA warning advised that JAKi should not be used in people with cardiovascular risk factors if there is a suitable treatment alternative available, such as a biologic.

Other studies have examined the characteristics and risk profile of people who initiated JAKi prior to regulatory warnings. In a UK registry study in rheumatoid arthritis, which included data on smoking status, 80% of 1341 initiators of JAKi had at least one risk factor for cardiovascular disease [49]. A US analysis of targeted therapy use in people with rheumatoid arthritis found 26% of those who were prescribed JAKi and 26% of those prescribed TNFi had cardiovascular or cancer risk factors [50]. People with ulcerative colitis, Crohn's disease and atopic dermatitis who initiate JAKi are younger than those with rheumatoid arthritis and may have reduced risk [51, 52, 53, 54, 55]. The mechanisms by which JAKi may affect cardiovascular disease and cancer are unknown, and the extent to which rheumatoid arthritis confers a background risk is also unknown [56]. It will be critical to continue to monitor these risk profiles across all indications as prescribing patterns change in response to regulatory warnings.

Similar to other studies, such as JAK‐pot, our analysis described JAKi as a class but most of the data in our study period relates to tofacitinib use. There is some evidence from real‐world studies that upadacitinib is associated with higher persistence compared to tofacitinib or baricitinib [12, 57]. Upadacitinib specifically inhibits JAK1, which may confer theoretical safety advantages compared to tofacitinib and baricitinib, which inhibit JAK1/3 and JAK2/3, respectively, which may be more likely to affect mediators associated with vascular risk [58]. As JAKi, and upadacitinib in particular, become available for more indications, it will be necessary to monitor the safety profile of JAKi as a class as well as the different JAKi medicines.

Our analysis benefitted from use of a national and representative sample; however, there are some important limitations. Firstly, the indication for prescribing is not directly available in the PBS 10% sample, instead we used specific PBS item numbers as proxies. Private dispensings are not included in the PBS 10% sample, although this is likely to have a minimal effect because of the high cost of private JAKi prescriptions. The PBS 10% sample does not include information on two of the four risk factors listed in the TGA warning, namely, smoking status and increased risk of cancer. In the JAK‐pot study, approximately half of new users had ever smoked [10]. Smoking and age over 65 years were considered major risk factors in the ORAL Surveillance study [45]. The validity of inferring indications from PBS item codes for targeted therapies is uncertain since formulations available for more than one indication may have multiple item codes. We anticipate the impact of this issue is likely to be small. Prior to October 2019, rheumatoid arthritis was the only indication for JAKi. After October 2019, it is theoretically possible that a formulation with an item code for rheumatoid arthritis may have been dispensed to a person with a different indication, but rheumatoid arthritis is more prevalent than any of the other indications for JAKi. In our data, 75% to 90% of PBS item codes were prescribed by a specialty consistent with the indications for those PBS item codes.

## 5 Conclusions

In conclusion, we showed that use of JAKi has increased over time likely due to the expanded indications for JAKi and new JAKi becoming listed on the PBS for subsidy. Almost half of new users initiated JAKi as their first‐line targeted therapy and almost half had at least one risk factor listed in the TGA warning. There will be a need for continued monitoring of JAKi use to evaluate whether the risk factor profile of new users changes in response to the TGA warning, and to monitor the longer‐term effectiveness and safety of JAKi use.

## Funding

This research was supported by the National Health and Medical Research Council Medicines Intelligence Centre of Research Excellence (ID: 1196900) and a Seed Grant from the School of Population Health, Faculty of Medicine and Health, UNSW Sydney. Dr. Falster is supported by a Heart Foundation Future Leader Fellowship (ID: 105609).

## Ethics Statement

Our study was approved by the NSW Population and Health Services Research Ethics Committee (Approval Number: 2019/ETH01176) and data access was granted by the Australian Government Services Australia External Request Evaluation Committee (Approval Number: RMS3250). Direct access to the data and analytical files to other individuals or authorities is not permitted without the express permission of the approving human research ethics committees and data custodians.

## Conflicts of Interest

Sallie‐Anne Pearson, Nicole L. Pratt, and David Liew are members of the Drug Utilisation Sub Committee of the Pharmaceutical Benefits Advisory Committee. The views expressed in this paper do not represent those of the Committee. Geoffrey O. Littlejohn has been involved in educational meetings and research studies supported by Abbvie Pty Ltd. in the last 3 years. Peter Youssef has received honoraria for lectures and consulting from AbbVie, Eli Lilly, Celltrion, Pfizer, Sandoz, Janssen, Astra Zeneca and Novarits. The other authors declare no conflicts of interest.

## Supporting information


**Figure S1:** Graphical representation of study design.
**Figure S2:** Quarterly temporal trends in the initiation of JAKi in Australia restricted to PBS item codes for rheumatoid arthritis, showing stabilisation of initiation rates from 2019 as JAKi were subsidised for new indications. Note that after 2019 it is possible that some of these item codes for rheumatoid arthritis were dispensed to people with other indications due to PBS listing for other conditions.
**Figure S3:** Quarterly trends in JAKi uptake with an alternative definition of b/tsDMARD‐naïve (no prior b/tsDMARD within 5 years prior).
**Figure S4:** Kaplan–Meier analysis of persistence to JAKi to 365 days stratified by JAKi medicine.
**Figure S5:** Kaplan–Meier analysis of persistence to JAKi to 365 days stratified by age group.
**Table S1:** ATC and PBS codes for JAKi medicines described in this study.
**Table S2:** Specialty of prescribers of PBS item codes.
**Table S3:** ATC codes for anti‐inflammatory medicines described in this study.
**Table S4:** ATC codes and PBS item codes for antiviral medicines.
**Table S5:** ATC codes used to identify medicines prescribed for comorbidities.
**Table S6:** Estimated periods of exposure for specific formulations of JAKi defined by PBS item codes.
**Table S7:** Characteristics of people with different indications initiating JAKi.
**Table S8:** Sensitivity analysis to test an alternative definition of treatment cessation.
**Table S9:** JAKi persistence stratified by JAKi medicine and by age.

## Data Availability

The PBS 10% sample was provided by the Australian Government Services Australia under licence. Access to these data by other individuals or authorities is not permitted without the express permission of the approving human research ethics committees and data custodians.
