# Supplementary material for: Janus Kinase Inhibitor Use Among People With Inflammatory Disease in Australia
Source: Pharmacoepidemiol Drug Saf. 2026 Oct 30;35(11):e70503. doi: 10.1002/pds.70503 (PMC13722534; doi:10.1002/pds.70503)
Supplement: Supplementary file 1 [file PDS-35-e70503-s001.docx]

**SUPPLEMENTARY MATERIAL**

**JANUS KINASE INHIBITOR USE AMONG PEOPLE WITH INFLAMMATORY DISEASE IN AUSTRALIA**

Claire T. Deakin,^1^ Michael O. Falster,^1^ Geoffrey O. Littlejohn,^2^ Peter Youssef,^3,4,5^ David Liew,^6,7,8^ Nicole L. Pratt,^9*^ Sallie-Anne Pearson^1*^

1. Medicines Intelligence Centre of Research Excellence, School of Population Health, Faculty of Medicine and Health, UNSW Sydney, Sydney, Australia
2. Department of Medicine, Monash University, Clayton, VIC, Australia
3. Royal Prince Alfred Hospital, Sydney, NSW, Australia
4. Faculty of Medicine and Health, University of Sydney, Sydney, NSW, Australia
5. Institute for Musculoskeletal Health, Sydney Local Health District, Sydney, NSW, Australia
1. Department of Rheumatology, Austin Health, Heidelberg, Victoria, Australia
2. Department of Clinical Pharmacology and Therapeutics, Austin Health, Heidelberg, Victoria, Australia
3. Department of Medicine, University of Melbourne, Parkville, Victoria, Australia
4. Quality Use of Medicines and Pharmacy Research Centre, Clinical and Health Sciences, University of South Australia, Adelaide, Australia

* *These authors contributed equally to this work*

Corresponding author:	Dr Claire T. Deakin

Address:	School of Population Health, Faculty of Health and Medicine, UNSW Sydney, Sydney, Australia

Email:	c.deakin@unsw.edu.au

**Supplementary Figure S1.** Graphical representation of study design

**Supplementary Figure S2.** Quarterly temporal trends in the initiation of JAKi in Australia restricted to PBS item codes for rheumatoid arthritis, showing stabilisation of initiation rates from 2019 as JAKi were subsidised for new indications. Note that after 2019 it is possible that some of these item codes for rheumatoid arthritis were dispensed to people with other indications due to PBS listing for other conditions.

**Supplementary Figure S3.** Quarterly trends in JAKi uptake with an alternative definition of b/tsDMARD-naïve (no prior b/tsDMARD within 5 years prior)

**Supplementary Figure S4.** Kaplan-Meier analysis of persistence to JAKi to 365 days stratified by JAKi medicine.

**Supplementary Figure S5.** Kaplan-Meier analysis of persistence to JAKi to 365 days stratified by age group.

**Supplementary Table S1. ATC and PBS codes for JAKi medicines described in this study**

| **JAKi** | **ATC code** | **Indication** | **Date available on the PBS** | **PBS item codes** |
| --- | --- | --- | --- | --- |
| Tofacitinib | L04AA29 | Rheumatoid arthritis | October 2015 | 10511F, 10517M, [12720L](https://www.pbs.gov.au/medicine/item/12720l) |
|  |  | Psoriatic arthritis | October 2019 | 11675L, 11690G |
|  |  | Ulcerative colitis | July 2021 | [12556W](https://www.pbs.gov.au/medicine/item/12556w), [12557X](https://www.pbs.gov.au/medicine/item/12557x), [12588M](https://www.pbs.gov.au/medicine/item/12588m)*, [12589N](https://www.pbs.gov.au/medicine/item/12589n)* |
| Baricitinib | L04AA37 | Rheumatoid arthritis | September 2018 | 11437Y, 11442F, 11443G, 11447L, 12708W, 12730B |
| Upadacitinib | L04AA44 | Rheumatoid arthritis | May 2020 | 11979L, 11989B, [12685P](https://www.pbs.gov.au/medicine/item/12685p), [12835M](https://www.pbs.gov.au/medicine/item/12835m), [13250J](https://www.pbs.gov.au/medicine/item/13250j), [13256Q](https://www.pbs.gov.au/medicine/item/13256q) |
|  |  | Psoriatic arthritis | July 2021 | [12624K](https://www.pbs.gov.au/medicine/item/12624k), [12648Q](https://www.pbs.gov.au/medicine/item/12648q) |
|  |  | Ankylosing spondilitis | July 2021 | [12621G](https://www.pbs.gov.au/medicine/item/12621g), [12625L](https://www.pbs.gov.au/medicine/item/12625l) |
|  |  | Atopic dermatitis | January 2022 | [12827D](https://www.pbs.gov.au/medicine/item/12827d), [12828E](https://www.pbs.gov.au/medicine/item/12828e), [12829F](https://www.pbs.gov.au/medicine/item/12829f), [12831H](https://www.pbs.gov.au/medicine/item/12831h), [12836N](https://www.pbs.gov.au/medicine/item/12836n) |
|  |  | Ulcerative colitis | May 2023 | [13249H](https://www.pbs.gov.au/medicine/item/13249h), [13262B](https://www.pbs.gov.au/medicine/item/13262b), [13265E](https://www.pbs.gov.au/medicine/item/13265e) |

*PBS item codes for the high -dose formulation of tofacitinib (10 mg twice daily)

**Supplementary Table S2. Specialty of prescribers of PBS item codes**

|  | **Item codes** | **Rheumatology** | **Dermatology** | **Gastroenterology** | **General medicine** | **Immunology** | **Other or not recorded** |
| --- | --- | --- | --- | --- | --- | --- | --- |
| **Ankylosing spondylitis** | [12621G](https://www.pbs.gov.au/medicine/item/12621g), [12625L](https://www.pbs.gov.au/medicine/item/12625l) | 34 (77.3%) | <6 (<10%) | 0 | <6 (<10%) | 6 (13.6%) | <6 (<10%) |
| **Atopic dermatitis** | [12827D](https://www.pbs.gov.au/medicine/item/12827d), [12828E](https://www.pbs.gov.au/medicine/item/12828e), [12829F](https://www.pbs.gov.au/medicine/item/12829f), [12831H](https://www.pbs.gov.au/medicine/item/12831h), [12836N](https://www.pbs.gov.au/medicine/item/12836n) | <6 (<5%) | 92 (88.5%) | 0 | <6 (<5%) | <6 (<5%) | <6 (<5%) |
| **Psoriatic arthritis** | 11675L, 11690G, [12624K](https://www.pbs.gov.au/medicine/item/12624k), [12648Q](https://www.pbs.gov.au/medicine/item/12648q) | 272 (72.7%) | 0 | 0 | 19 (5.1%) | 21 (5.6%) | 62 (16.6%) |
| **Rheumatoid arthritis** | 10511F, 10517M, [12720L](https://www.pbs.gov.au/medicine/item/12720l), 11437Y, 11442F, 11443G, 11447L, 12708W, 12730B, 11979L, 11989B, [12685P](https://www.pbs.gov.au/medicine/item/12685p), [12835M](https://www.pbs.gov.au/medicine/item/12835m), [13250J](https://www.pbs.gov.au/medicine/item/13250j), [13256Q](https://www.pbs.gov.au/medicine/item/13256q) | 1734 (75.7%) | 13 (0.6%) | <6 (<1%) | 136 (5.9%) | 52 (2.3%) | 351 (15.3%) |
| **Ulcerative colitis** | [12556W](https://www.pbs.gov.au/medicine/item/12556w), [12557X](https://www.pbs.gov.au/medicine/item/12557x), [12588M](https://www.pbs.gov.au/medicine/item/12588m), [12589N](https://www.pbs.gov.au/medicine/item/12589n), [13249H](https://www.pbs.gov.au/medicine/item/13249h), [13262B](https://www.pbs.gov.au/medicine/item/13262b), [13265E](https://www.pbs.gov.au/medicine/item/13265e) | <6 (<3%) | 0 | 87 (94.6%) | <6 (<3%) | 0 (0%) | <6 (<3%) |

**Supplementary Table S3. ATC codes for anti-inflammatory medicines described in this study**

| **Class** | **Medicine** | **ATC code** |
| --- | --- | --- |
| TNFi | Adalimumab | L04AB04  L04AA17 |
|  | Certolizumab pegol | L04AB05 |
|  | Etanercept | L04AB01  L04AA11 |
|  | Golimumab | L04AB06 |
|  | Infliximab | L04AB02  L04AA12 |
| Other targeted therapy | Abatacept | L04AA24 |
|  | Guselkumab | L04AC16 |
|  | Ixekizumab | L04AC13 |
|  | Ozanimod | L04AA38 |
|  | Secukinumab | L04AC10 |
|  | Tocilizumab | L04AC07 |
|  | Ustekinumab | L04AC05 |
|  | Vedolizumab |  |
| Immunosuppressants | Methotrexate | L01BA01  L04AX03 |
|  | Hydroxychloroquine | M01CA  P01BA02 |
|  | Chloroquine | P01BA01 |
|  | Sulfazalazine | A07EC01 |
|  | Leflunomide | M01AX  L04AA13 |
|  | Mycophenolate | L04AA06 |
|  | Tacrolimus | L04AA05  L04AD02 |
|  | Azathioprine | L01BB01 |
|  | Ciclosporin | L04AD01 |
|  | Mercaptopurine | L01BB02 |
|  | Mesalazine | A07EC02 |
|  | Balsalazide | A07EC04 |
|  | Olsalazine | A07EC03 |
| Corticosteroids | Prednisone | H02AB07 |
|  | Prednisolone | H02AB06  S01BA04  S01BB  C05AA04  A07EA01  S03BA02 |
|  | Methylprednisolone | D07AC14  H02AB04 |
|  | Dexamethasone | H02AB02  S01BA01  S02CA06 |
|  | Budesonide | R03BA02 |
| NSAIDs | Indometacin | M01AB01 |
|  | Sulindac | M01AB02 |
|  | Diclofenac | M01AB05  D11AX18  S01BC03 |
|  | Diclofenac; combinations | M01AB55 |
|  | Parecoxib | M01AC01 |
|  | Meloxicam | M01AC06 |
|  | Ibuprofen | M01AE01 |
|  | Naproxen | M01AE02 |
|  | Ketoprofen | M01AE03 |
|  | Tiaprofenic acid | M01AE11 |
|  | Indometacin | M01AE51  M01AB01 |
|  | Mefenamic acid | M01AG01 |
|  | Celecoxib | M01AH01 |
|  | Lumiracoxib | M01AH06 |
|  | Acetylsalicylic acid (aspirin) | N02BA01 |
|  | Diflunisal | N02BA11  M01AX |

**Supplementary Table S4. ATC codes and PBS item codes for antiviral medicines**

| **Antiviral medicine** | **ATC code** | **PBS item code** |
| --- | --- | --- |
| Aciclovir | 05AB01 | 1003T, 1007B, 1052J, 1555W |
| Valaciclovir | J05AB11 | 5480K, 8134D, 6280M, 9568N, 8064K, 8133C |
| Famciclovir | J05AB09 | 2274Q, 8002E, 8092X, 8217L, 8896F, 8897G |

**Supplementary Table S5. ATC codes used to identify medicines prescribed for comorbidities**

Reproduced from Pratt et al 2018.^1^

| **Rx-risk comorbidity category** | **ATC codes** | **Weights for Rx-Risk Score** |
| --- | --- | --- |
| Alcohol dependency | N07BB01–N07BB99 | 6 |
| Allergies | R01AC01–R01AD60, R06AD02–R06AX27, R06AB04 | -1 |
| Anticoagulants | B01AA03–B01AB06, B01AE07, B01AF01, B01AF02, B01AX05 | 1 |
| Antiplatelets | B01AC04–B01AC30 | 2 |
| Anxiety | N05BA01–N05BA12, N05BE01 | 1 |
| Arrhythmia | C01AA05, C01BA01–C01BD01, C07AA07 | 2 |
| Benign prostatic hyperplasia | G04CA01–G04CA99, G04CB01, G04CB02* | 0 |
| Bipolar disorder | N05AN01 | -1 |
| Chronic airways disease | R03AC02–R03DC03, R03DX05 | 2 |
| Congestive heart failure | C03DA02–C03DA99, C07AB02—if PBS item code is 8732N, 8733P, 8734Q, 8735R, C07AB07, C07AG02, C07AB12, C03DA04 (C03CA01–C03CC01 and C09AA01–C09AX99, C09CA01–C09CX99)† | 2 |
| Dementia | N06DA02–N06DA04, N06DX01 | 2 |
| Depression | N06AA01–N06AG02, N06AX03–N06AX11, N06AX13–N06AX18, N06AX21–N06AX26 | 2 |
| Diabetes | A10AA01–A10BX99 | 2 |
| Epilepsy | N03AA01–N03AX99 | 0 |
| Glaucoma | S01EA01–S01EB03, S01EC03–S01EX99 | 0 |
| Gastrooesophageal reflux disease | A02BA01–A02BX05 | 0 |
| Gout | M04AA01–M04AC01 | 1 |
| Hepatitis B | J05AF08, J05AF10, J05AF11 | NA |
| Hepatitis C | J05AB54, L03AB10, L03AB11, L03AB60, L03AB61, J05AE14, J05AE11–J05AE12, J05AX14, J05AX15, J05AX65, J05AB04 | NA |
| HIV | J05AE01–J05AE10, J05AF12–J05AG05, J05AR01–J05AR99, J05AX07–J05AX09, J05AX12, J05AF01–J05AF07, J05AF09 | 0 |
| Hyperkalaemia | V03AE01 | 4 |
| Hyperlipidaemia | A10BH03‡, C10AA01–C10BX09 | -1 |
| Hypertension | C03AA01–C03BA11, C03DB01, C03DB99, C03EA01, C09BA02–C09BA09, C09DA02–C09DA08, C02AB01–C02AC05, C02DB02–C02DB99 (C03CA01–C03CCO1 or C09CA01–C09CX99)§ |  |
| Hyperthyroidism | H03BA02, H03BB01 | 2 |
| Hypothyroidism | H03AA01–H03AA02 | 0 |
| Irritable bowel syndrome | A07EC01–A07EC04, A07EA01–A07EA02, A07EA06, L04AA33 | 0 |
| Ischaemic heart disease: angina | C01DA02–C01DA14, C01DX16, C08EX02 | 2 |
| Ischaemic heart disease: hypertension | C07AA01–C07AA06, C07AA08–C07AB01, C07AB02—if PBS item code is not 8732N, 8733P, 8734Q, 8735R, C07AG01, C08CA01–C08DB01, C09DB01–C09DB04, C09DX01, C09BB02–C09BB10, C07AB03, C09DX03, C10BX03¶ | -1 |
| Incontinence | G04BD01–G04BD99 | 0 |
| Inflammation/pain | M01AB01–M01AH06 | -1 |
| Liver failure | A06AD11, A07AA11 | 3 |
| Malignancies | L01AA01–L01XX41 | 2 |
| Malnutrition | B05BA01–B05BA10 | 0 |
| Migraine | N02CA01–N02CX01 | -1 |
| Osteoporosis/Paget’s | M05BA01–M05BB05, M05BX03, M05BX04, G03XC01, H05AA02 | -1 |
| Pain | N02AA01–N02AX02, N02AX06, N02AX52, N02BE51 | 3 |
| Pancreatic insufficiency | A09AA02 | 0 |
| Parkinson’s disease | N04AA01–N04BX02 | 3 |
| Psoriasis | D05AA01–D05AA99, D05BB01 D05BB02, D05AX02, D05AC01–D05AC51, D05AX52 | 0 |
| Psychotic illness | N05AA01–N05AB02, N05AB06–N05AL07, N05AX07–N05AX13 | 6 |
| Pulmonary hypertension | C02KX01–C02KX05, PBS item code 9547L, 9605M | 6 |
| Renal disease | B03XA01–B03XA03, A11CC01–A11CC04, V03AE02, V03AE03, V03AE05 | 6 |
| Smoking cessation | N07BA01–N07BA03, N06AX12 | 6 |
| Steroid-responsive disease | H02AB01–H02AB10 | 2 |
| Transplant | L04AA06, L04AA10, L04AA18, L04AD01, L04AD02 | 0 |
| Tuberculosis | J04AC01–J04AC51, J04AM01–J04AM99 | NA |

*Benign prostatic hyperplasia medicines are tested for gender—must be male. Females suffering from bladder obstructions can be prescribed medicines used to treat benign prostatic hyperplasia.

†Must have at least two medicines prescribed with one of those medicines having an ATC code from C03CA01–C03CC01 and the other having an ATC code from either C09AA01–C09AX99 or C09CA01–C09CX99.

‡Combination product for hyperlipidaemia and diabetes.

§Can have medicine dispensed with an ATC code C03CA01–C03CC01 or C09AA01–C09AX99, but not both, as this would indicate chronic heart failure.

¶Combination product for hyperlipidaemia and ischaemic heart disease: hypertension.

N/A, not applicable.

**Supplementary Table S6. Estimated periods of exposure for specific formulations of JAKi defined by PBS item codes**

| **JAKi** | **ATC code** | **Indication** | **PBS item code** | **Estimated period of exposure (days)** |
| --- | --- | --- | --- | --- |
| Tofacitinib | L04AA29 | Rheumatoid arthritis | 10511F | 34 |
|  |  |  | 10517M | 34 |
|  |  |  | [12720L](https://www.pbs.gov.au/medicine/item/12720l) | 30 |
|  |  | Psoriatic arthritis | 11675L | 34 |
|  |  |  | 11690G | 34 |
|  |  | Ulcerative colitis | [12556W](https://www.pbs.gov.au/medicine/item/12556w) | 30 |
|  |  |  | [12557X](https://www.pbs.gov.au/medicine/item/12557x) | 35 |
|  |  |  | [12588M](https://www.pbs.gov.au/medicine/item/12588m) | 34 |
|  |  |  | [12589N](https://www.pbs.gov.au/medicine/item/12589n) | 34.5 |
| Baricitinib | L04AA37 | Rheumatoid arthritis | 11437Y | 32 |
|  |  |  | 11442F | 32 |
|  |  |  | 11443G | 34 |
|  |  |  | 11447L | 33 |
|  |  |  | 12708W | 28.5 |
|  |  |  | 12730B | 32.5 |
| Upadacitinib | L04AA44 | Rheumatoid arthritis | 11979L | 34 |
|  |  |  | 11989B | 33 |
|  |  |  | [12685P](https://www.pbs.gov.au/medicine/item/12685p) | 31.5 |
|  |  |  | [12835M](https://www.pbs.gov.au/medicine/item/12835m) | 36 |
|  |  |  | [13250J](https://www.pbs.gov.au/medicine/item/13250j) | 31 |
|  |  | Psoriatic arthritis | [12624K](https://www.pbs.gov.au/medicine/item/12624k) | 34 |
|  |  |  | [12648Q](https://www.pbs.gov.au/medicine/item/12648q) | 34 |
|  |  | Ankylosing spondilitis | [12621G](https://www.pbs.gov.au/medicine/item/12621g) | 35 |
|  |  |  | [12625L](https://www.pbs.gov.au/medicine/item/12625l) | 34 |
|  |  | Atopic dermatitis | [12827D](https://www.pbs.gov.au/medicine/item/12827d), | 33 |
|  |  |  | [12828E](https://www.pbs.gov.au/medicine/item/12828e) | 34 |
|  |  |  | [12829F](https://www.pbs.gov.au/medicine/item/12829f) | 31 |
|  |  |  | [12831H](https://www.pbs.gov.au/medicine/item/12831h) | 34 |
|  |  |  | [12836N](https://www.pbs.gov.au/medicine/item/12836n) | 29.5 |
|  |  | Ulcerative colitis | [13249H](https://www.pbs.gov.au/medicine/item/13249h) | 33 |
|  |  |  | [13265E](https://www.pbs.gov.au/medicine/item/13265e) | 14 |

**Supplementary Table S7. Characteristics of people with different indications initiating JAKi**

|  | **Rheumatoid arthritis (n=2,290)** | **Psoriatic arthritis (n=374)** | **Ankylosing spondylitis (n=44)** | **Ulcerative colitis (n=92)** | **Atopic dermatitis (n=104)** |
| --- | --- | --- | --- | --- | --- |
| Sex (n, %) | | | | | |
| Female | 1,711 (74.7%) | 265 (70.9%) | 33 (75%) | 41 (44.6%) | 44 (42.3%) |
| Male | 579 (25.3%) | 109 (29.1%) | 11 (25%) | 51 (55.4%) | 60 (57.7%) |
| Age at initiation, years (median, IQR) | 60 [51 - 69] | 55 [45 - 63] | 55 [42 - 60] | 44 [32 - 54] | 45 [28 - 58] |
| Age group at initiation (n, %) | | | | | |
| 18-34 years | 111 (4.8%) | 31 (8.3%) | 7 (15.9%) | 27 (19.6%) | 39 (37.5%) |
| 35-44 years | 205 (9.0%) | 60 (16.0%) | 6 (13.6%) | 21 (22.8%) | 13 (12.5%) |
| 45-54 years | 444 (19.4%) | 87 (23.3%) | 8 (18.2%) | 22 (27.2%) | 17 (16.3%) |
| 55-64 years | 674 (29.4%) | 115 (30.7%) | 17 (38.6%) | 8 (13%) | 21 (20.2%) |
| 65-74 years | 585 (25.5%) | 59 (15.8%) | 6 (13.6%) | <10 (10.9%) | 7 (6.7%) |
| 75-84 years | 245 (10.7%) | <25 (<6.7%) | 0 (0%) | <6 (6.5%) | <6 (<5.8%) |
| 85 years and older | 26 (1.1%) | <6 (<1.6%) | 0 (0%) | <6 (<1.5%) | <6 (<5.8%) |
| JAKi medicine of initiation (n, %) | | | | | |
| Tofacitinib | 1,233 (53.8%) | 281 (75.1%) | 0 (0%) | 92 (100%) | 0 (0%) |
| Baricitinib | 417 (18.2%) | 0 (0%) | 0 (0%) | 0 (0%) | 0 (0%) |
| Upadacitinib | 640 (27.9%) | 93 (24.9%) | 44 (100%) | 0 (0%) | 104 (100%) |
| Initiation of high dose tofacitinib, 10 mg twice daily (n, %) | 0 (0%) | 0 (0%) | 0 (0%) | 72 (78.3%) | 0 (0%) |
| Prescriber speciality of first JAKi | | | | | |
| Rheumatology | 1,734 (75.7%) | 272 (72.7%) | 34 (77.3%) | <6 (<3.0%) | <6 (<3.0%) |
| Gastroenterology | <6 (<0.5%) | 0 (0%) | 0 (0%) | 87 (94.6%) | 0 (0%) |
| Dermatology | <15 (<1.0%) | 0 (0%) | <6 (<13.6%) | 0 (0%) | 92 (88.5%) |
| Immunology | 52 (2.3%) | 21 (5.6%) | 6 (13.6%) | 0 (0%) | <6 (<6.0%) |
| General medicine | 136 (5.9%) | 19 (5.1%) | <6 (<13.6%) | <6 (<6.0%) | <6 (<3.0%) |
| Other or not recorded | 351 (15.3%) | 62 (16.6%) | <6 (<13.6%) | <6 (<6.0%) | <6 (<6.0%) |
| Concomitant PBS medicine use at JAKi initiation (n, %) | | | | | |
| None | 93 (4.1%) | 35 (9.4%) | 8 (18.2%) | 9 (9.8%) | 22 (21.2%) |
| Immunosuppressant | 1,874 (81.8%) | 260 (69.5%) | 11 (25%) | 74 (80.4%) | 33 (31.7%) |
| Corticosteroid | 1,677 (73.2%) | 236 (63.1%) | 26 (59.1%) | 63 (68.5%) | 66 (63.5%) |
| NSAID | 1,055 (46.1%) | 193 (51.6%) | 27 (61.4%) | 7 (7.6%) | 14 (13.5%) |
| Number of unique targeted therapies received up to 365 days prior (n, %) | | | | | |
| 0 | 1,017 (44.4%) | 136 (36.4%) | 15 (34.1%) | 30 (32.6%) | 98 (94.2%) |
| 1 | 1,008 (44%) | 179 (47.9%) | 20 (45.5%) | 48 (52.2%) | <6 (<6.0%) |
| 2 | 231 (10.1%) | 49 (13.1%) | <10 (<20.0%) | 9 (9.8%) | <6 (<6.0%) |
| ≥3 | 34 (1.5%) | 10 (2.7%) | <6 (<13.6%) | <6 (<6.0%) | 0 (0%) |
| Class of most recent targeted therapies received within 365 days prior (n, %) | | | | | |
| TNFi | 781 (34.1%) | 135 (36.1%) | 21 (47.7%) | 29 (31.5%) | <6 (<6.0%) |
| Other biologic | 492 (21.5%) | 103 (27.5%) | 8 (18.2%) | 33 (35.9%) | <6 (<6.0%) |
| Medicine use for comorbidities up to 365 days prior (n, %) |  |  |  |  |  |
| Cardiovascular disease or risk (at least one of the following)  Hypertension  Hyperlipidaemia  Anticoagulants  Antiplatelets | 681 (29.7%)  500 (21.8%)  651 (28.4%)  205 (9.0%)  95 (4.1%) | 91 (24.3%)  67 (17.9%)  91 (24.3%)  17 (4.5%)  9 (2.4%) | 13 (29.5%)  11 (25%)  15 (34.1%)  <6 (<13.6%)  <6 (<13.6%) | 15 (16.3%)  9 (9.8%)  15 (16.3%)  <10 (<10.0%)  <6 (<3.0%) | 13 (12.5%)  8 (7.7%)  13 (12.5%)  <6 (<3.0%)  <6 (<3.0%) |
| Diabetes | 212 (9.3%) | 32 (8.6%) | <6 (<13.6%) | <6 (<6.0%) | 6 (5.8%) |
| Hepatitis B, Hepatitis C, HIV or Tuberculosis | 113 (4.9%) | 18 (4.8%) | <6 (<13.6%) | 6 (6.5%) | 10 (9.6%) |
| Malignancies | 7 (0.3%) | <6 (<3.0%) | <6 (<13.6%) | 15 (16.3%) | 0 (0%) |
| Renal disease | 14 (0.6%) | 0 (0%) | 0 (0%) | <6 (<6.0%) | 0 (0%) |
| Anxiety, Bipolar Disorder, Depression or Psychotic Illness | 864 (37.7%) | 168 (44.9%) | 18 (40.9%) | 16 (17.4%) | 36 (34.6%) |
| Rx-Risk Comorbidity Index (median, IQR) | 3 [1 - 5] | 3 [1 - 5] | 3 [1 - 4] | 2 [1 - 4] | 2 [0 - 4] |
| Aged over 65 years and medicine use for cardiovascular disease or risk | 406 (17.7%) | 22 (5.9%) | <6 (<13.6%) | <6 (<6.0%) | 6 (5.8%) |

**Supplementary Table S8. Sensitivity analysis to test an alternative definition of treatment cessation**

| **People persistent to JAKi at 365 days** | **Treatment gap defined as 3x estimated period of exposure** | **Treatment gap defined as 2x estimated period of exposure** |
| --- | --- | --- |
| Whole cohort of JAKi initators (n=2,904) | 1,735 (59.7%) | 1,734 (59.7%) |
| JAKi initiators naïve to prior targeted therapy (n=1,296) | 820 (63.3%) | 819 (63.2%) |

**Supplementary Table S9.** JAKi persistence stratified by JAKi medicine and by age

|  | **JAKi initiators persisting to 365 days (n=2,904)** |
| --- | --- |
| JAKi medicine  Tofacitinib  Baricitinib  Upadacitinib | 886 (55.2%)  260 (62.4%)  589 (66.9%) |
| Age  18-34 years  35-44 years  45-54 years  55-64 years  65-74 years  75 years or older | 114 (53.0%)  176 (57.0%)  328 (56.7%)  526 (63.0%)  412 (61.9%)  179 (58.7%) |

**References**

1.	Pratt NL, Kerr M, Barratt JD, et al. The validity of the Rx-Risk Comorbidity Index using medicines mapped to the Anatomical Therapeutic Chemical (ATC) Classification System. *BMJ Open*. 2018;8(4):e021122. doi:10.1136/BMJOPEN-2017-021122
